# Assessment of Indoor Air Quality Problems in Office-Like Environments: Role of Occupational Health Services

**DOI:** 10.3390/ijerph15040741

**Published:** 2018-04-12

**Authors:** Paolo Carrer, Peder Wolkoff

**Affiliations:** 1 Department of Biomedical and Clinical Sciences “L. Sacco”, University of Milan, 20157 Milan, Italy; paolo.carrer@unimi.it; 2 National Research Centre for the Working Environment, Lersø Parkallé 105, 2100 Copenhagen Ø, Denmark

**Keywords:** comfort, indoor air quality, management, offices, sickness, symptoms

## Abstract

There is an increasing concern about indoor air quality (IAQ) and its impact on health, comfort, and work-performance in office-like environments and their workers, which account for most of the labor force. The Scientific Committee on Indoor Air Quality and Health of the ICOH (Int. Comm. Occup. Health) has discussed the assessment and management of IAQ problems and proposed a stepwise approach to be conducted by a multidisciplinary team. It is recommended to integrate the building assessment, inspection by walk-through of the office workplace, questionnaire survey, and environmental measurements, in that order. The survey should cover perceived IAQ, symptoms, and psychosocial working aspects. The outcome can be used for mapping the IAQ and to prioritize the order in which problems should be dealt with. Individual health surveillance in relation to IAQ is proposed only when periodical health surveillance is already performed for other risks (e.g., video display units) or when specific clinical examination of workers is required due to the occurrence of diseases that may be linked to IAQ (e.g., Legionnaire’s disease), recurrent inflammation, infections of eyes, respiratory airway effects, and sensorial disturbances. Environmental and personal risk factors should also be compiled and assessed. Workplace health promotion should include programs for smoking cessation and stress and IAQ management.

## 1. Introduction

The indoor air quality (IAQ) in office-like environments is an issue of increasing focus because office workers provide services of high relevance. The office workers, which account for most of the labor force in many countries, are occupationally exposed to biological, chemical, physical, ergonomic, and psycho-logical/social loads with a potentially high and diversified impact on comfort [1], work-related health problems [2], sickness absence [3], and risk of deteriorated work performance [4,5]. Another issue is an ageing workforce, later retirement, and the general trend (in Europe) that the workforce in public offices is being reduced due to economic constraints. In this context, effects of IAQ on health, well-being, and work-performance have been reported in office-like environments in recent decades (e.g., [5,6,7]).

Modern offices are built with new materials, equipment, and the use of a variety of cleaning, consumer, and personal care products; their emission of chemicals and particles reflects IAQ together with the incoming outdoor air. New energy saving strategies, like lightning, heating, cooling, and ventilation, also impact the perception of IAQ. Furthermore, pollutants that are emitted from office equipment—e.g., laser printer emissions (ozone, primary VOCs, and particles)—and secondary VOCs derived from reactive indoor air chemistry may be of concern [8,9,10,11].

The EnVIE project prioritized the following diseases caused or exacerbated by poor IAQ: allergic and asthma symptoms; chronic obstructive and pulmonary diseases; airborne respiratory infections; cardiovascular mortality and morbidity; lung cancer; odor and sensory irritation in eyes and airways (Sick Building Syndrome (SBS) symptoms) [12]; for this terminology, see below.

The term SBS has been used to define buildings in which an excessive prevalence of occupants (>20%) report one or more symptoms that may be associated with the IAQ and climate at work [13]. However, the SBS terminology, apart from being semantically misleading, today is considered obsolete [9,14,15,16], simply because no single pollution load is causative of all SBS symptoms; rather, focus is on the individual specific symptomatology and the identification of potential risk factors for specific symptoms or signs [9,17]. For instance, Brightman et al. (2008) concluded that “these findings (questionnaires in buildings at large; added by authors) imply that it is counterproductive to dichotomize buildings into healthy vs. unhealthy; instead the prevalence of health problems related to buildings span a continuum” [14]. Furthermore, the neuropsychological symptoms (e.g., headache, fatigue) “may in fact stem, at least partially, from a different origin” than exposure (added by the authors) [18]. Thus, specific symptoms or exacerbation thereof may be associated with one or more risk factors and possibly in a concerted and amplifying manner.

Health effects potentially related to exposure to indoor air pollutants in office environments include acute and semi-acute effects and longer-term based effects. The former can be divided into immediate perceived IAQ that is related to odor perception (olfactorius nerve) and semi-acute effects that are characterized by some latency, like sensory irritation in eyes and airways (trigeminus nerve), and symptoms related to the central nervous system (e.g., headache and fatigue) [15,19].

Many epidemiological studies have addressed IAQ and associated exposure related symptoms in office workers [6,14,18,20,21,22]. For instance, one recent study showed that eye symptoms were associated with proximity to outdoor pollution like traffic, portable humidifiers, and crowded spaces [23], for further discussion, see [17]. Other risk factors include visual display unit work and technical causes such as inadequate ventilation [24], low humidity [25], or high temperature, especially regarding eye symptoms [17]. Further, mold in moisture-damaged buildings is another exposure risk that may deteriorate the IAQ and impact susceptible people, e.g., with asthma symptoms [26,27].

The office type also plays a role in the prevalence of symptom reporting and sickness absence [3]; generally, the more crowded the more dissatisfaction and increase of adverse health effects [28,29,30,31]. It should be noted, however, that factors like noise, distance from window, and privacy also may influence the overall perception of IAQ and the prevalence of symptoms.

Indoor symptoms have also been related to psychosocial environments and mental stress at work [2]. For instance, an association between overall comfort, indoor symptoms, high efforts and low rewards, the mood scale, and self-estimated productivity has been observed in a large survey in modern EU office buildings [6]. Consequently, there is an interest in exploring potential synergies between psychosocial stress and indoor air on health and comfort of office workers [20,32,33,34]. It is essential to acknowledge how odor annoyance—e.g., caused by degraded building materials [35] or generated from moisture-damage (MVOCs)—may influence general comfort; psychic reactions with nonspecific symptoms are plausible, but they would not induce direct physiological reactions like sensory irritation in eyes and upper airways [27,36]. Typical symptoms after exposure to unpleasant odors are fatigue, impaired power of concentration, headache, nausea, and insomnia, including possible mood disorders [27]. Furthermore, susceptible people—i.e., with high distress (low affectivity) or being diagnosed with asthma or multiple diagnoses of asthma/allergy—may react more intensely than people with low distress or without asthma [37,38].

Studies in office buildings have focused primarily on acute health symptoms: unspecific symptoms of irritation of eyes, nose and mucous membranes, fatigue, and headache experienced by office worker. Longer-term effects due to continuous or repeated exposure to indoor air pollutants may be associated with aggravation of asthma exacerbation and allergic responses, oxidative stress and inflammation, chronic obstructive and pulmonary disease, lung cancer, and cardiovascular diseases. These effects are generally not evaluated in office workers due to the complexity and required resources for clinical testing, which seem unrealistic in view of occupants’ mobility and the inhomogeneity of exposures [7].

Several experimental studies have demonstrated deteriorated workers’ performance caused by poor IAQ—e.g., text processing [5]—but, also by general conditions of the office—e.g., lighting and noise [39]. A complete understanding of the cause(s)–effect relationships and type of loss of work performance is complex. Chemical pollutants and bioeffluents, rather than carbon dioxide itself, have been suggested to cause mental distraction and stress by perceived poor IAQ [40], but other mechanisms are also in play [40,41]. For instance, Maula et al. demonstrated a weak loss in cue-utilization, but not for other performance tests by low ventilation as a metric for bioeffluents in a typical office environment [42]. The overall concern about work performance is not only related to absenteeism and associated diseases due to the work environment (IAQ), but it may result from less comfortable working conditions, e.g. inadequate IAQ [5,43,44] and low relative humidity [17,25].

## 2. Occupational Health Services

Occupational health services are increasingly requested to assess the risk and monitor the health status of workers in non-industrial building sectors. This topic has been discussed by experts of the Italian Society of Occupational Health [45], during a special session at the Indoor Air 2008 Conference [46], and by the members of the Scientific Committee on Indoor Air Quality and Health of the International Commission on Occupational Health (ICOH) starting from the ICOH 2009 Conference [47]. The role of the occupational health services about assessment and management of IAQ problems in office-like environments has been proposed as shown below.

### 2.1. Collaboration in Risk Assessment—Risk Management

A multidisciplinary team approach with experts in occupational medicine, IAQ, building physics, and (airway) toxicology, is recommended for risk assessment of IAQ problems. It is recommended to integrate the building assessment, questionnaire survey, and environmental measurements, in that order. This should also include a survey of office equipment, e.g., printers, cleaning procedures, and the use of cleaning products and of other consumer products.

In group surveys the contribution of the multidisciplinary team could be as follows:

▪ Information meeting at the company site with the safety committee and staff responsible for ventilation, maintenance, working environment consultants, and/or the local working environment authority (labor inspection); the primary goal is to identify the type of IAQ or climate problem(s) reported and prioritized in the workplace evaluation, and to obtain information about the building.
▪ Inspection by walk-through of the company workplace.
▪ Planning of future activities:
  - Questionnaire survey (see below) and assessment thereof.
  - Collaboration at the technical building assessment, decision about IAQ measurements if required, and evaluation of the results. It is recommended to postpone IAQ measurements if some action—e.g., intervention—is planned. Measurements, in general, should only be carried out in case of suspected strong sources of pollution or other relevant climatic parameters, or if it is necessary to control that air quality parameters are within regulatory standards/guidelines.
  - Individual clinical examination if required (see below).
▪ Evaluation of the combined results and definition of the risk management activities.

### 2.2. Questionnaire to Assess Workers’ Comfort

The diagnosis of problem buildings/offices is not at individual level in the traditional medical sense, but rather a diagnosis at group level with the implementation of a questionnaire survey. In larger companies (minimum 45 employees), a questionnaire survey may provide a good overview. The questionnaire should cover questions about the perceived IAQ and climate parameters, symptoms, and working aspects. The role of personal factors like use of medication and psychosocial stress—e.g., affectivity—are important issues that may influence the individual response and perception of health, should be considered [44,48]. Psychological factors may also influence the outcome of a questionnaire response [49]. It has been hypothesized that psychosocial stressors could influence the reporting of individuals becoming more susceptible [33]; consequently, the psychosocial parameters should be examined when risk factors of health and comfort are investigated.

Standardized and translated questionnaires for indoor climate cases are available, for instance the Örebro model and MM questionnaire [50]. Note that the response rate strongly depends on the design of the questionnaire, the recall period, and the neutrality of letter of invitation. It is essential that the questionnaires are treated anonymously. The company’s safety representative or committee can perform distribution and collection of questionnaires. Alternatively, the questionnaire could be an online survey. Such surveys are ideal for monitoring the impact of intervention (i.e., before and after); it should be noted, however, that at least one later survey is advisable to establish stability and avoid the first impression reactions and filter out the inter alia the influence of different mood reactions.

It has been suggested that building-related health complaints should be investigated at the work-area level and not at a building wide level. An occupant-centric medical evaluation should guide environmental investigations, especially when screening results of indoor environmental and air quality measurements show that the work areas in the building are within regulatory standards and air quality guidelines [4].

An important role of the multidisciplinary team is the analysis and interpretation of the results. The questionnaire survey data can be used for mapping the perceived IAQ, indoor climate, and the psychosocial aspects and to prioritize the order in which the problems should be tackled, e.g., measurements. However, two important issues should be considered. First, to distinguish whether the reported symptoms are specifically associated with the working place and not with other external stimuli—e.g., home, transport, or nearby industry. Further, it has been argued that reverse causality may prevail as confirmed with ‘dummy’ questions; thus, the chicken and the egg situation should be considered when identifying associations [51]. Second, it should be realized, both on a group and individual level, that reported symptoms in some cases are borderline with actual disease (in low to moderate degree) and not directly caused by the IAQ, although some symptoms may be exacerbated by the IAQ.

## 3. Health Surveillance

Individual health surveillance in relation to IAQ problems is proposed only when periodical health surveillance is already performed for other risks (e.g., video display units). Furthermore, individual clinical examination of workers is also required when the occurrence of symptoms (signs) appears to be associated with inadequate IAQ (e.g., Legionnaire’s disease), infection of the eyes, respiratory (and inflammatory) effects in upper and lower airways, and sensorial disturbances. The examination of individuals must encompass:

- Workplace description, that includes building and offices, ventilation conditions, amount of available (floor) space per office worker, lighting, standard of cleaning, visible moisture damages, and/or mold growth; furthermore, the thermal climate is important, like experience of cold feet, cold/warm air, dry or stuffy air, smells, static electricity, and noise.
- Description of symptoms (and signs), with special regard to mucous membrane (sensory) irritation, skin and general symptoms, and relationship with work (symptom development). When do symptoms appear during the week; are symptoms present on working days and/or days off, and seasonal variation; do colleagues report similar symptoms. Routine physical examination, focusing on eyes, nose, throat, skin, and lungs.
- Allergy (atopy) evaluation, when indicated; for example, in case of asthma and/or rhinitis, where allergens are suspected at the workplace, e.g., mold growth, animal fur, and plants.
- Other risk factors; for instance, transport conditions, use of medication, and use of contact lenses.
- Furthermore, it should be considered that the worker may have a disease or unrecognized disease that influences the overall perception of the IAQ and symptom reporting.

## 4. Promotion of Health

Health promotion is performed at various locations. Worksites are among the settings that should receive special attention. WHO states that the workplace “has been established as one of the priority settings for health promotion into the 21st century” because it influences “physical, mental, economic and social well-being” and “offers an ideal setting and infrastructure to support the promotion of health of a large audience”. Workplace health promotion should include programs for smoking cessation (important source of IAQ pollution), stress (synergies between psychosocial stress and indoor air on health and comfort of office workers), and on adequate measures for IAQ management. Furthermore, it is considered highly appropriate, when moving from an old to new workplace, to provide neutral and trustworthy information about the IAQ.

## 5. Conclusions

There is an increasing demand for (occupational health) services to assess work related risks and monitor the health status of office workers. A multidisciplinary team of experts in occupational medicine, IAQ, building physics, and toxicology, is recommended for the assessment and management of IAQ problems. A questionnaire survey is recommended as a first step, while expensive physical, chemical, and microbiological measurements should only be carried out in case of an anticipated action or strong suspicion to specific agents causing the problem or if it is required to ensure that air quality parameters are within regulatory standards/guidelines.

Guidance about the role of the occupational health service in the assessment and management of IAQ problems has been provided and includes collaboration in risk assessment, use of a questionnaire survey for comfort and health evaluation of the workers, health surveillance when already performed for other risks or in case of specific clinical examinations and health promotion.

Further, synergies with other risk factors—e.g., psychosocial stress—may potentially also be important and need to be evaluated, too.
